# Apolipoprotein A-IV Has Bi-Functional Actions in Alcoholic Hepatitis by Regulating Hepatocyte Injury and Immune Cell Infiltration

**DOI:** 10.3390/ijms24010670

**Published:** 2022-12-30

**Authors:** Wan-Hong Li, Li Zhang, Yue-Ying Li, Xin-Yue Wang, Jin-Liang Li, Shu-Ning Zhao, Ming-Qi Ni, Qian Li, Hui Sun

**Affiliations:** 1 Pharmaceutical Experiment Teaching Center, College of Pharmacy, Harbin Medical University, Harbin 150081, China; 2 Pharmaceutical Analysis and Analytical Chemistry, College of Pharmacy, Harbin Medical University, Harbin 150081, China

**Keywords:** alcoholic hepatitis, apolipoprotein A-IV, inflammation, immune cell infiltration, CIDEC

## Abstract

Alcohol abuse can lead to alcoholic hepatitis (AH), a worldwide public health issue with high morbidity and mortality. Here, we identified apolipoprotein A-IV (APOA4) as a biomarker and potential therapeutic target for AH. APOA4 expression was detected by Gene Expression Omnibus (GEO) databases, Immunohistochemistry, and qRT-PCR in AH. Bioinformatics Methods (protein–protein interaction (PPI) network, Gene Ontology (GO) and Kyoto Encyclopedia of Genes and Genomes (KEGG) pathways and Gene Set Enrichment Analysis (GSEA) were used to show down-stream gene and pathways of APOA4 in AH. AML-12 cells were used to evaluate the biological function of APOA4 using an ELISA kit (AST, ALT, and IL-1β) and flow cytometry (ROS activity). Both in vivo and in vitro, APOA4 expression was significantly elevated in the AH model induced by alcohol (ETOH). AML-12 cell damage was specifically repaired by APOA4 deficiency, while AST, ALT, and IL-1β activity that was increased by ETOH (200 µmol, 12 h) were suppressed. APOA4 inhibition increased intracellular ROS induced by ETOH, which was detected by flow cytometry. Functional and PPI network analyses showed Fcgamma receptor (FCGR) and platelet activation signaling were potential downstream pathways. We identified *CIDEC* as a downstream gene of APOA4. The CIDEC AUC values for the ROC curves were 0.861. At the same time, APOA4 silencing downregulated the expression of *CIDEC*, whereas the knockdown of *CIDEC* did not influence the expression of APOA4 in AML-12 cells. Collectively, APOA4 regulates *CIDEC* expression and immune cell infiltration and may hold great potential as a biomarker and therapeutic target for AH.

## 1. Introduction

Alcohol abuse can lead to alcoholic liver disease (ALD), which is the main cause of liver-related morbidity and mortality worldwide and contributes to 0.9% of all fatalities worldwide [1,2,3]. About 35% of heavy alcohol users may develop alcoholic hepatitis (AH) associated with high mortality [4,5]. Currently, the diagnosis of AH depends mainly on pathologic examination and medical imaging. However, finding a reliable biomarker and therapeutic target for AH is necessary. 

Patients with AH are highly predisposed to infection due to diminished antimicrobial responses in monocytes and neutrophils [6]. Immunotherapy is one of the most advanced therapies to date and has been widely used in the treatment of AH [7,8,9]. Recent investigations have linked the immune microenvironment and the invasion of immune cells to AH onset, progression, prognosis, and treatment response. Both immune cells and stroma cells play crucial roles in hepatic biology. Immune-associated molecules modulate the invasion of immune cells, a process associated with the response to immune therapy. Hence, we speculated that immune-associated factors might be used to estimate the prognosis and treatment for patients with AH. 

APOA4, a plasma lipoprotein, modulates several metabolic processes, such as the metabolism of lipids and glucose [10]. It has been reported that APOA4 accelerates the secretion of triglycerides from the liver [11]. The content of APOA4 in plasma correlates positively with the level of high-density lipoproteins; APOA4 significantly promotes insulin secretion triggered by glucose and decreases the generation of products in the liver. By stimulating pancreatic insulin production, APOA4 improves glucose homeostasis, which contributes to the suppression of hepatic gluconeogenesis and the promotion of glucose uptake into adipose tissues in the absence of insulin [12]. APOA4 is markedly associated with obesity in mice, humans, and type 2 diabetes [13]. In addition, the level of circulating APOA4 has been used as a biomarker for the early diagnosis of liver fibrosis [14,15,16]. It has become increasingly clear that APOA4 is involved in various physiological functions, including inflammation reduction [17] and antioxidant activity [18]. Furthermore, many diverse transcription factors, including ERR-α, HNF-4α, CREB, SERBP1, CREBH, and PPARα, could influence APOA4 expression [19]. These factors slowed the progress of the AH [20,21,22]. However, the underlying molecular regulatory mechanisms and cascades of APOA4’s role in AH remain unclear.

In the current study, we demonstrated that APOA4, an immune-associated gene, has remarkable prognostic value and anti-AH effect. We also developed a model to estimate the response to therapy of people with AH.

## 2. Results

### 2.1. Validation of APOA4 Expression

**Figure 1:**
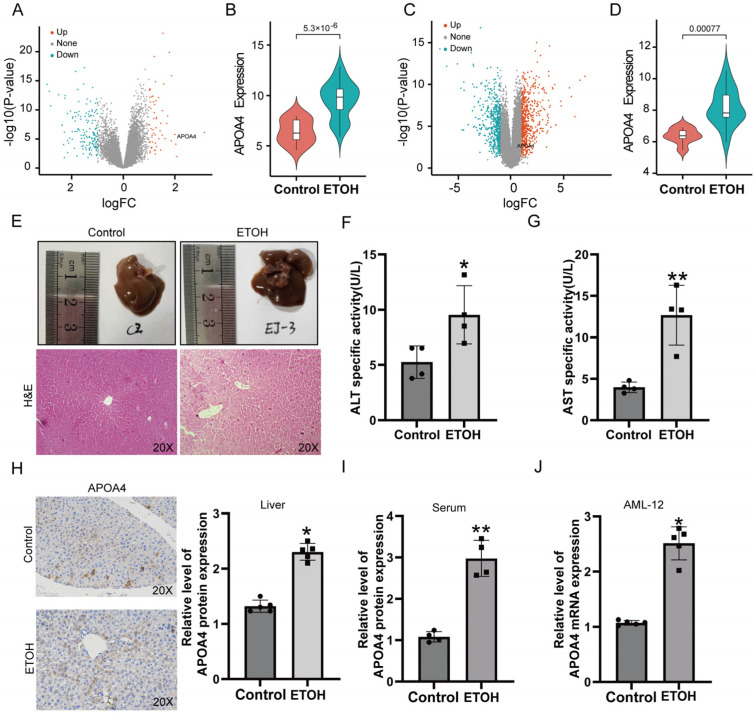
APOA4 is elevated in AH. Volcano plots showed the differentially expressed genes (DEGs) between AH and normal liver tissues in the E-MTAB-2664 dataset and the GSE28619 dataset. Orange dots indicate the up-regulated genes, blue dots indicate down-regulated genes, and gray dots indicate non-DEGs. (**A**,**B**) Volcano map and Box plot of APOA4 expression between AH and normal liver tissues in the E-MTAB-2664 dataset. (**C**,**D**) Volcano map and Box plot of APOA4 expression between AH and normal liver tissues in the GSE28619 dataset. (**E**) Hematoxylin-eosin (H&E) staining. (**F**,**G**) The activity of ALT and AST. (**H**,**I**) The expression of APOA4 in AH tissue as well as serum. (**J**) The expression of APOA4 in AML-12 cells after 200 μmol/L ETOH treatment for 12 h. (*n* = 3–6) * *p* < 0.05, ** *p* < 0.01 vs. the control group.

Our analysis indicated differential expression of APOA4 in both the Array Express (logFC = 3.149, *p* < 0.0001) and GEO (logFC = 1.838, *p* = 0.0008) cohorts (Figure 1A,B). APOA4 expression was significantly higher in the patients with AH (Figure 1C,D). To further confirm the role of APOA4 in AH, we established a vivo model of AH (Figure 1E). Our data showed that the alanine aminotransferase (ALT)/aspartate aminotransferase (AST) in serum is significant elevation in the alcohol hepatocyte (ETOH) group (Figure 1F,G). While in both AH tissue and serum, after treatment with alcohol, the expression of APOA4 was increased (Figure 1H,I). In addition, in the vitro model [23], AML-12 cells were treated for 12 h with alcohol at a concentration of 200 μmol/L. The expression of APOA4 was significantly increased in the ETOH group (Figure 1J). These data indicated that APOA4 was elevated in AH.

### 2.2. Alcohol-Induced AML-12 Cell Injury in the Presence of APOA4 Knockdown

**Figure 2:**
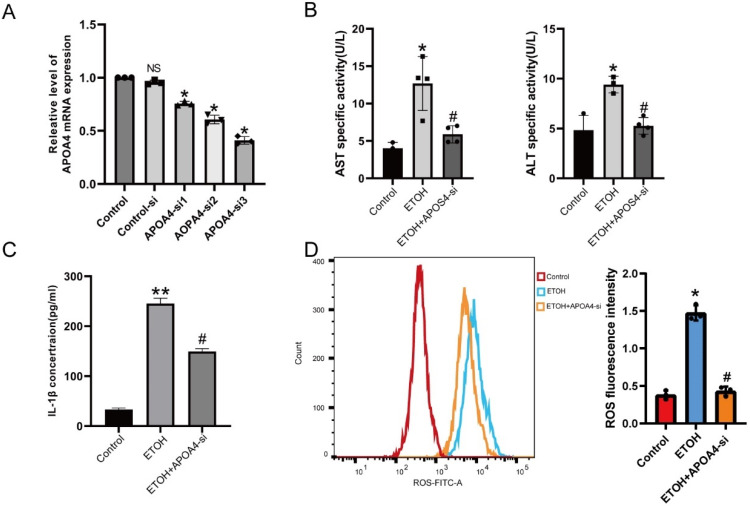
APOA4 inhibition decreased cell injury in ETOH -treated AML-12 cells. (**A**) AML-12 cells were transfected with APOA4 siRNA or control siRNA for 48 h. The expression of APOA4 was determined with qRT-PCR. (**B**) The AST and ALT activity in AML-12 cells after treatment with APOA4-si stimulated by ETOH or left untreated. (**C**) IL-1β activity by IL-1β ELISA kit. (**D**) ROS fluorescence intensity was detected by flow cytometry. (*n* = 3–6) * *p* < 0.05, ** *p* < 0.01 vs. the control group; # *p* < 0.05 vs. the ETOH group or ^NS^ *p* > 0.05 vs. the Control group.

To explore the role of APOA4 in AH, we knocked down the expression of APOA4 in AML-12 cells (Figure 2A). The ETOH treatment increased the activity of AST, ALT (Figure 2B), and IL-1β (Figure 2C), whereas APOA4 knockdown decreased AST, ALT, and IL-1β activity. DCFH-DA staining result also indicated increased intracellular ROS in the ETOH group. Silenced APOA4 notably inhibited ROS increasing induced by ETOH (Figure 2D). Therefore, our data showed that suppression of APOA4 could protect against ETOH-induced AML-12 injury.

### 2.3. Identification of APOA4 Co-Expressed Genes

**Figure 3:**
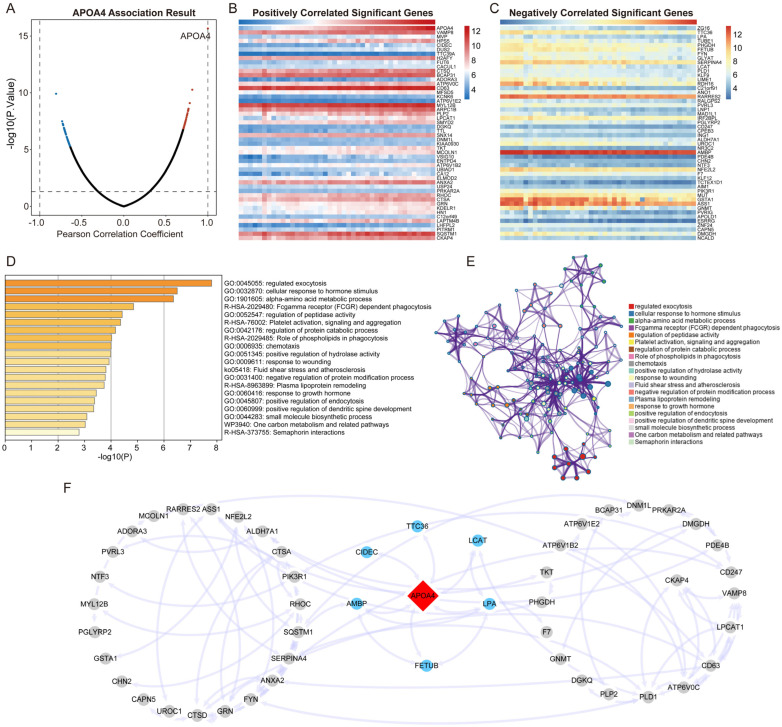
Functional enrichment and PPI analysis of APOA4 co-expressed genes. (**A**) A Pearson test was used to analyze the correlation between APOA4 and other genes in AH. Red indicates positively correlated genes; blue indicates negatively correlated genes. (**B**,**C**) Heat maps illustrating genes positively and negatively correlated with APOA4 in AH (top 50). (**D**) Analysis of Gene Ontology and Kyoto Encyclopedia of Genes and Genomes pathways associated with APOA4 expression. (**E**) Network plot of the relationships among the top 20 terms. (**F**) PPI network plot of genes co-expressed with APOA4.

To investigate the biological significance of APOA4 in AH and the downstream pathways involved. We identified the protein-coding genes of APOA4 and its co-expression genes in AH. As shown in Figure 3A, Pearson’s correlation analysis was used to identify genes that showed a positive and negative correlation with APOA4 in AH tissues from the ArrayExpress cohort. The heatmap demonstrated the expression levels of the top 50 protein-coding genes that positively and negatively correlated with APOA4, respectively (Figure 3B,C).

### 2.4. Functional Enrichment and PPI Analysis

In this study, the Metascape database was used to determine the potential biological functions of APOA4. These co-expression genes were mainly enriched in pathways such as Fcgamma receptor (FCGR) dependent phagocytosis, platelet activation signaling, and aggregation. They were primarily involved in biological processes such as the regulation of exocytosis, alpha-amino acid metabolism, and negative regulation of the protein modification process (Figure 3D,E). These results suggest that the pathways of phagocytosis, amino acid metabolism, and protein modification are involved in the lesions of AH, which was strongly related to APOA4 expression. Furthermore, STRING tools were employed to analyze the interaction between APOA4 and protein-coding genes mentioned earlier. Figure 3F illustrates the results of the analysis.

### 2.5. Hub Gene Recognition and Validation

We created a PPI network to uncover the intrinsic relationships among the genes co-expressed with APOA4. Six genes (*AMBP*, *CIDEC*, *FETUB*, *LCAT*, *LPA*, and *TTC36*) were identified as hub genes because they were found to be directly associated with APOA4.

**Figure 4:**
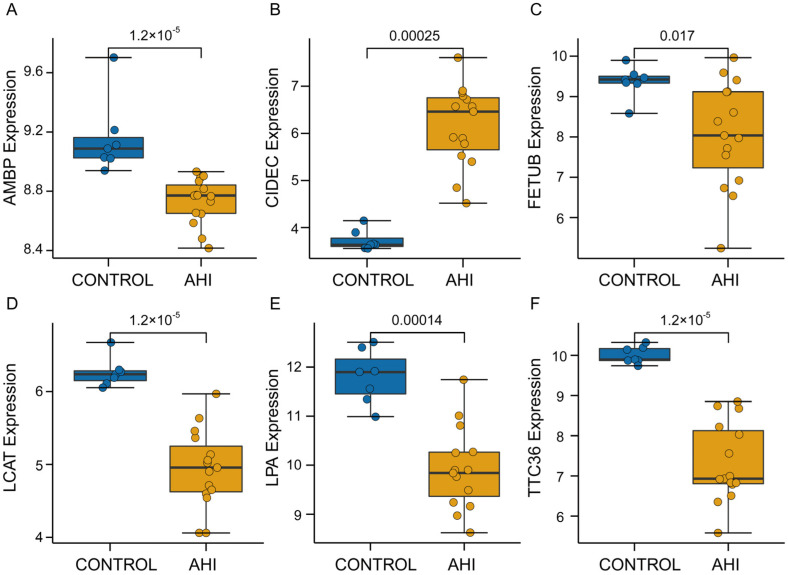
Validation of hub genes. (**A**) *AMBP*, (**B**) *CIDEC*, (**C**) *FETUB*, (**D**) *LCAT*, (**E**) *LPA,* and (**F**) *TTC36*.

**Figure 5:**
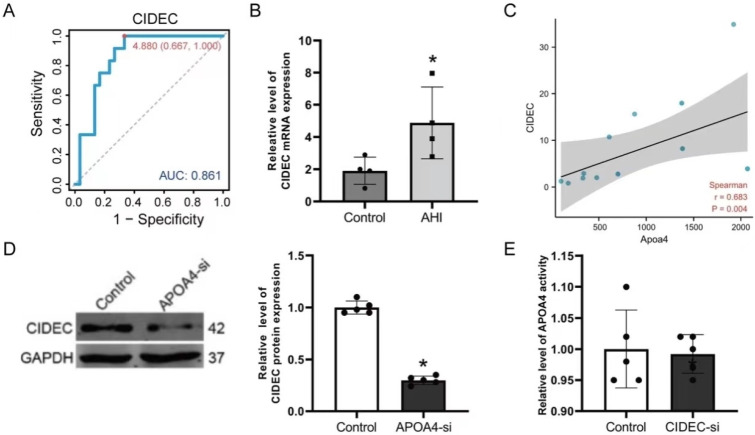
The role of CIDEC in AH. (**A**) CIDEC, in the ArrayExpress cohort. (**B**) The expression of *CIDEC* in AH tissue compared with the control group. (**C**) The correlation of *CIDEC* and APOA4 in AH liver tissue. The correlated concentration between APOA4 and *CIDEC* was determined by western blotting (**D**) and qRT-PCR (**E**). (*n* = 3–6) * *p* < 0.05, vs. the Control group.

Next, we validated these hub genes in the GEO cohort. As shown in Figure 4A–F, *AMBP*, *FETUB*, *LCAT*, *LPA*, and *TTC36* were all significantly overexpressed in normal liver tissues, whereas only *CIDEC* was overexpressed considerably in AH tissues. Furthermore, we plotted the receiver operating characteristic (ROC) curves for the six hub genes in the ArrayExpress cohort based on the expression of genes with the corresponding disease states. The six hub genes had excellent diagnostic value in discriminating between AH and control samples, with an AUC of 0.836 for *AMBP*, 0.861 for *CIDEC*, 0.850 for *FETUB*, 0.883 for *LCAT*, 0.811 for *LPA*, and A 0.889 for *TTC36* (Figure 5A and Supplementary Figure S1). To test the relationship between APOA4, *CIDEC,* and other hub genes, we used qRT-PCR to analyze liver expressions after treating ETOH in mice. *CIDEC* was up-regulated in ETOH-treated mouse livers (Figure 5B and Supplementary Figure S2). Simultaneously, CIDEC was found to be positively correlated with APOA4 in both normal liver tissues and hepatocellular carcinoma tissues (Supplementary Figure S3). We then tested the relationship between APOA4 and *CIDEC* in AH liver tissue using by Spearman test. Our data showed APOA4 positively correlated with *CIDEC* (r = 0.628, *p* = 0.004, Figure 5C). On the other hand, we also analyzed the expression of *CIDEC* after the knockdown of APOA4 in AML-12 cells. At the same time, we first observed the effect of the *CIDEC* on AML-12 cells’ injury by ETOH (Figure S4A–D). Our data showed *CIDEC* knockdown could inhibit AML-12 cell injury after treatment with ETOH. The *CIDEC* protein was significantly down-regulated by APOA4, but APOA4 mRNA levels remained unchanged after treatment with *CIDEC*-si in AML-12 cells (Figure 5D,E).

### 2.6. Relationship between APOA4 and Immune Infiltrating Cells in AH

**Figure 6:**
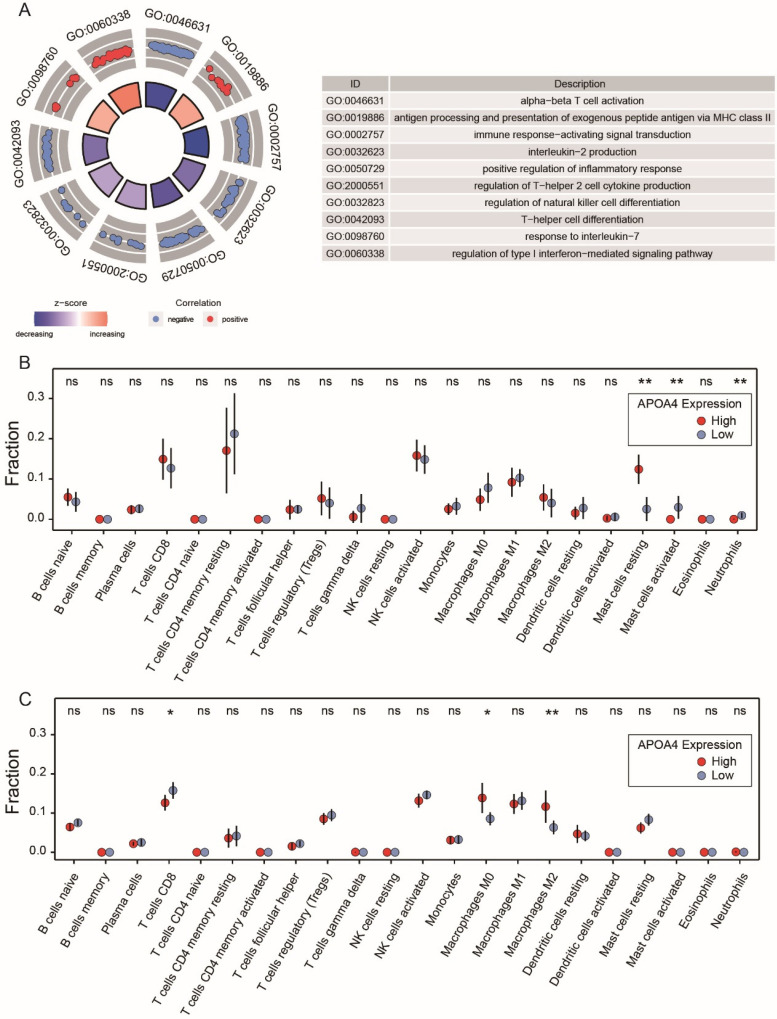
Proportions of 22 immune infiltrating cells’ subpopulations in normal liver tissues and AH tissues were associated with APOA4. (**A**) Analysis of Gene Ontology associated with APOA4 expression by GSEA enrichment analysis. (**B**) Immune infiltrating cells in normal liver tissues. (**C**) Immune infiltrating cells in AH tissues. * *p* < 0.05, ** *p* < 0.01 vs. the high group, ^ns^ *p* > 0.05 vs the high group.

To elucidate the mechanism underlying APOA4’s involvement in the pathological progression of AH, we first used GSEA enrichment analysis to show the potential signaling pathways of APOA4 in AH. The data displayed in Figure 6A shows that APOA4 was connected with alpha–beta T cell activation, immune response-activating signal transduction, positive regulation of inflammatory response, and regulation of T-helper 2 cell cytokine production signaling pathways. These findings highlighted the importance of the immune system in the abnormal expression of APOA4 in AH. Based on the expression profiles from the ArrayExpress cohort, we assessed the relationship between the APOA4 expression and the immune system in 22 types of immune-invading cells via the CIBERSORT algorithm. As illustrated in Figure 6B, resting mast cells (*p* = 0.005) increased in the high APOA4 group of normal liver tissues, whereas activated neutrophils (*p* = 0.006) and mast cells (*p* = 0.010) decreased. In AH tissues, while M0 (*p* = 0.016) and M2 (*p* = 0.003) macrophages were enriched in the high APOA4 group, and resting mast cells (*p* = 0.026), CD8 T cells (*p* = 0.021), and follicular helper T cells (*p* = 0.022) were enriched in the low APOA4 group (Figure 6C). We also observed the relationship between *CIDEC* and immune infiltrating cells in AH and found similar results as APOA4 in immune infiltrating cells in AH (Supplementary Figure S5).

## 3. Discussion

AH is the most severe type of ALD and is associated with high mortality [24]. Heavy alcohol consumption alters lipid metabolism, resulting in lipid aggregation in hepatocytes and the secretion of danger-associated molecular patterns, which, in combination with gut-originating pathogen-associated molecular patterns, trigger an inflammatory response characteristic of AH [25,26]. AH is usually associated with ETOH’s misuse, progressive jaundice, and inflammatory liver injury [23]. Since AH progresses as time goes on, early diagnosis, potential therapeutic targets screened, and downstream pathways have significant clinical importance [27]. Unfortunately, there are few non-invasive, dependable methods for detecting alcohol-induced hepatocyte injury and alcoholic hepatitis.

APOA4 is a highly conserved protein that often exists in eukaryotic cells [28]. It is primarily located in the nucleus; however, it can shuttle from the nucleus to the cytoplasm and subsequently function in nucleocytoplasmic signal transport [29,30,31]. Previous studies found that APOA4 levels were markedly elevated in cancer and growing cells, in contrast to quiescent cells [32,33]. Over-expression of APOA4 promotes the growth and proliferation of diverse cancer cell types [34]. All of these implied that APOA4 might be a prospective target for cancer gene treatment [35].

Nevertheless, few investigations on APOA4 in AH have been conducted. Here, we first find that APOA4 is up-regulated in the tissue of AH patients, and APOA4 can regulate immune infiltrating cells in AH. These results show that APOA4 may be a potential biomarker and therapeutic target in AH. To test this hypothesis, we used molecular biological methods to explore the effect of APOA4 on AH. Our data showed that silenced APOA4 significantly decreased the AML-12 cell injury induced by ETOH, and APOA4 inactivated the ROS activity induced by ETOH. These data indicate that APOA4 contributed to the anti-AH effect.

What is the molecular mechanism involved in APOA4 in AH? Xu et al. found *CIDEC* could promote the development of alcoholic steatohepatitis in mice and humans [36]. We found *CIDEC* may be a gene downstream of APOA4 in AH with a high AUV value (0.861) (Figure 4, Figure 5, and Figures S1–S3). To further confirm the downstream gene of APOA4 in AH, we employed molecular Biotechnology (western blot, ELISA kit, and flow cytometry) to detect the effect of *CIDEC* in AML-12 cells and AH tissues, and results showed *CIDEC* was up-regulated in ETOH-treated liver cells and positively correlated with APOA4 in AH. Furthermore, the expression of *CIDEC* modulates lipid deposition and secretion, both of which are significant steps in AH [37], thus confirming our hypothesis.

In addition, APOA4 has an immune-regulatory role in some diseases, such as in alveolar macrophages [38], spondylarthritis [39], as well as obesity-associated inflammatory hepatic steatosis [40]. The result showed APOA4 was connected with immune-relative signaling pathways, such as alpha-beta T cell activation, immune response-activating signal transduction, positive regulation of inflammatory response, and T-helper 2 cell cytokine production signaling pathways (Figure 6A). The innate immune responses triggered by pathogen-associated molecular patterns, such as the translocation of gut microflora and the sterile danger signals emanating from hepatocytes injured by alcohol, resulting in the activation of Kupffer cells (resident liver macrophages) and the mobilization and activation of macrophages in the liver [41,42,43]. Macrophages mobilize neutrophils to the site of liver injury, thus amplifying proinflammatory reactions and liver damage [44]. Nonetheless, these neutrophils have been documented to be mainly dysfunctional [45,46]. Because the activation of macrophages is critical in ALD onset, determining and characterizing the circulating biomarkers of macrophage activation in individuals with AH is essential. Although the diagnosis of AH is based on clinical and laboratory findings, novel biomarkers are required to estimate clinical outcomes and disease severity [47,48]. At the same time, dysregulating APOA4 in AH tissue and normal liver tissue regulates different immune cells. Such as, the resting mast cells are increased with high expression of APOA4 in normal liver tissues; otherwise, the activated neutrophils and mast cells are decreased. At the same time, M0 and M2 macrophages were enriched in the high APOA4 group in AH tissues, while the resting mast cells, CD8 T cells, as well as follicular helper T cells, were increased in the low expression APOA4 group (Figure 6C). These data showed the connection of APOA4 in immune cell infiltration in AH.

Above all, our data suggested that APOA4 has an antagonistic effect on immunity in patients with AH, thus providing new ideas and strategies for investigating APOA4 as an immunotherapy for AH. The APOA4/CIDEC/immune cell infiltration axis is expected to serve as a new biomarker or target, potentially leading to the development of the next generation of AH therapy.

## 4. Materials and Methods

### 4.1. Data Collection and Preprocessing

First, AH-related microarray data were obtained from the GEO data resource (www.ncbi.nlm.gov/geo, accessed on 3 October 2022) and the Array Express database (www.ebi.ac.uk/arrayexpress, accessed on 3 October 2022). The GSE28619 dataset contained data from seven people with normal livers and 15 patients with AH, whereas the E-MTAB-2664 dataset included data from 12 healthy individuals and 30 patients with AH. Two datasets were background connected and normalized with the “affy” package. We additionally downloaded the file that corresponded to the dataset to convert probes to gene symbols. For duplicate gene symbols, averaged overexpression values are reported. Data was accessed on 1 January 2021.

### 4.2. Identification of Genes Co-Expressed with APOA4 and Functional Enrichment Analysis

The top 50 genes with positive and negative Pearson’s correlation coefficients were selected as genes co-expressed with APOA4. Analysis with Metascape (http://metascape.org/gp/index.html#/main/step1, accessed on 3 October 2022), a free web analysis tool for gene functional enrichment analysis, revealed 100 co-expressed genes, which were imported into the Metascape data resource for Gene Ontology and Kyoto Encyclopedia of Genes and Genomes pathway analysis. Analysis with Metascape was accessed on 1 February 2021.

### 4.3. Protein-Protein Interaction (PPI) Analysis

We used the STRING data resource (https://string-db.org/, accessed on 3 October 2022) to explore the PPI network and Cystoscope v3.6.1 software to visualize this network. The cross-talk score was set at 0.4 in the STRING data resource. In the PPI network, genes directly interacting with APOA4 were considered hub genes and were used in subsequent analyses. STRING data resource was accessed on 1 February 2021.

### 4.4. Immune Infiltration Analysis

CIBERSORT, a deconvolution algorithm, calculated the proportion of 22 different types of immune cells in each tissue by analyzing the relative expression levels of 547 genes in individual tissue samples per their gene expression profiles [49]. The 22 types of immune cells include naive B cells, memory B cells, plasma cells, CD8+ T cells, naive CD4+ T cells, CD4+ resting memory T cells, CD4+ memory activated T cells, follicular helper T cells, regulatory T cells, γ δ T cells, resting natural killer cells, activated natural killer cells, monocytes, M0 macrophages, M1 macrophages, M2 macrophages, resting dendritic cells, activated dendritic cells, resting mast cells, activated mast cells, eosinophils, and neutrophils. Subsequently, we divided the samples into high and low-expression groups according to the medium APOA4 expression value in healthy and alcoholic liver tissue, respectively. Therefore, in our study, the CIBERSORT algorithm was applied to quantify the percentage of immune cells in the APOA4 high expression and the APOA4 low expression groups in the ArrayExpress cohort. Furthermore, the number of permutations of the default signature matrix was set to 100.

### 4.5. Animal Studies

Approximately 16 mice (male, ten weeks old) were obtained from Changsheng Biotechnology (Shenyang, China). The mice were randomly divided into two groups. One group was fed a nutritionally adequate liquid diet (Cat. #F1259, BIO-SERV, Flemington, NJ, USA) without ETOH pair-fed as control. The other was fed with a 5% ethanol (ETOH) diet as the alcohol-fed experimental group. The ethanol concentration for the ethanol diets in the experimental group gradually increased from 0% to 5% to acclimatize the mice to ethanol (Cat. #F1258, BIO-SERV, USA). The 5% ethanol and control diets were provided during the night for 12 h (20:00–8:00), and the normal diets were provided during daylight (8:00–20:00). All operations followed the manufacturer’s instructions throughout the experiment. In our study, all procedures were allowable according to the “Guide for the Care and Use of Laboratory Animals” published by the National Institutes of Health. Euthanasia was performed with cervical dislocation under anesthesia. Animal studies were approved by the Institutional Animal Care and Use Committee of Harbin Medical University, Harbin, China (Approval Code: IRB3015722). Sample analyses were not blinded. All tissues were collected for further study.

### 4.6. Cell Culture and Treatment

AML-12, a mouse hepatocyte cell line, was obtained from ShangCheng Beina ChuangLian Biology Technology Co., Ltd. (Shanghai, China). The cells were maintained under 37 °C and 5% CO_2_. The AML-12 cells were cultured in a 1:1 mixture of Dulbecco’s modified Eagle’s medium (cat. 11320033, Gibco, New York, NY, USA)/Ham’s F-12 medium (cat. 11320082, Gibco, New York, NY, USA), with 40 ng/mL dexamethasone, 5 μg/mL insulin-transferrin-selenium (cat. I1884, Sigma-Aldrich, St. Louis, SL, USA), and 10% fetal bovine serum (cat. FB65011, CLARK Bioscience, Australia).

Before further extraction, cells were pre-treated with 200 μmol/L ethanol or left untreated (control) for 12 h. An APOA4-small interfering RNA (APOA4-si) kit and RiboFECT CP Transfection Kit were obtained from RiboBio (Guangzhou, China). The RiboFECT CP Transfection Kit was used to transfect APOA4-si into AML-12 cells to down-regulate APOA4 expression. After transfection for 36 h, the AML-12 cells were treated with EtOH for 12 h or left untreated. After 48 h of treatment, cells were harvested for subsequent experiments, such as cell viability and biochemical analyses. The sequences of the APOA4 siRNA were as follows:

si-m-APOA4_001: 5′-GACCTGCAAGATCAGATCA-3′
si-m-APOA4_002: 5′-GCTGTAGAACAGTTTCAGA-3′
si-m-APOA4_003: 5′-GCAGCTGGAACAGTTCAGA-3′

### 4.7. Cell Viability

A Cell Counting, Kit-8 (Mei lune, Dalian, China), was used to analyze the viability of AML-12 cells. The cells were seeded in 96-well plates and incubated at 37 °C for two h. The optical density values were measured at 450 nm with a microplate reader (Thermo Fisher Multiskan FC, Shanghai, China).

### 4.8. Western Blotting

The cells were lysed with RIPA buffer and protein inhibitor (100:1, Beyotime, Shanghai, China). The total protein was detected with a BCA kit (Beyotime, Shanghai, China). Electrophoresis was then performed with 60–80 μg protein for approximately 1–2 h. The proteins were then transferred to an NC membrane for 2 h in an ice bath. The NC membrane was immediately placed into a solution with 5% non-fat milk and stored at room temperature for two hours to block nonspecific sites. Primary antibodies to the following proteins were used for western blotting: CIDEC (Abclonal, Wuhan, China) and β-actin (Abclonal, Wuhan, China).

### 4.9. ROS Level Assay

ROS production in AML-12 cells was assessed using 2′,7′-dichlorofluorescein diacetate (DCFH-DA) (Meilunebio, Dalian, China). The AML-12 cells were seeded into six-well plates at a density of 5 × 10^5^ cells per well and treated with ETOH or ETOH + APOA4-si for 48 h alone. A concentration of 10 mmol/L was achieved by diluting DCFH-DA with a serum-free medium by a factor of 1:1000. DCFH-DA was introduced after the cell culture media had been taken out. For 20 min, the cells were incubated at 37 °C. With media devoid of serum, the cells were washed three times. Flow cytometry (BD LSRFortessa, Becton Dickinson, Franklin Lakes, NJ, USA) was used to analyze ROS levels.

### 4.10. Enzyme-Linked Immunosorbent Assay (ELISA)

AML-12 cell supernatants from each subgroup were analyzed for interleukin (IL)-1β, AST (Aspartate transaminase), and ALT (Alanine transaminase). We used Quantikine ELISA Kits to determine IL-1β (Servicebio, Wuhan, China), AST (Elabscience Biotechnology Co., Ltd., Wuhan, China), and ALT (Elabscience Biotechnology Co., Ltd., Wuhan, China) activity.

### 4.11. RNA Isolation and Quantitative RT-PCR

A TRIzol reagent (Invitrogen, Shanghai, China) was used to extract total RNA from AML-12 cells. To reverse transcription of the total RNA, we used the First Strand cDNA Synthesis Kit (Thermo Fisher Scientific, Rockford, IL, USA) under previous conditions. Light Cycler Fast Start DNA Master Plus SYBR Green I Kit’s protocols (Roche Diagnostics, Burgess Hill, UK) were strictly followed during the RT-PCR. To measure APOA4 and CIDEC expression levels, GAPDH was used as an endogenous control. The 2^−ΔΔCt^ method was used for statistical analysis. 

Sequences of the primers are as follows:

APOA4-forward: 5′-GGCCTGAGGTAGGAGGTTGT-3′,
APOA4-reverse: 5′-CAGTGCGTGTCGTGGAGT-3′;
CIDEC-forward: 5′-AAATACTCTATGGCTGCCTCCCC-3′,
CIDEC-reverse: 5′-TGTCTAACAAGTCCCCAAACTGT-3′;
GAPDH-forward: 5′-CATGGGTGTGAACCATGAGA-3′,
GAPDH-reverse: 5′-GTCTTCTGGGTGGCAGTGAT-3′.

### 4.12. Statistical Analysis

Statistical analyses were implemented in R v.3.6.1 software. To compare differences between groups, the nonparametric Wilcoxon rank-sum test was used. Pearson’s correlation coefficients were used to explore the relationships among continual variables. Unless otherwise specified, *p* < 0.05 was considered to indicate statistical significance.

## Data Availability

The data that support this study are available within the article and available from the authors upon request.

## Supplementary Materials

The following supporting information can be downloaded at: https://www.mdpi.com/article/10.3390/ijms24010670/s1.

## References

[1] Singal A.K., Bataller R., Ahn J., Kamath P.S., Shah V.H. (2018). ACG Clinical Guideline: Alcoholic Liver Disease. Am. J. Gastroenterol..

[2] Rocco A., Compare D., Angrisani D., Sanduzzi Zamparelli M., Nardone G. (2014). Alcoholic disease: Liver and beyond. World J. Gastroenterol..

[3] Lamas-Paz A., Hao F., Nelson L.J., Vazquez M.T., Canals S., Gomez Del Moral M., Martinez-Naves E., Nevzorova Y.A., Cubero F.J. (2018). Alcoholic liver disease: Utility of animal models. World J. Gastroenterol..

[4] O’Shea R.S., Dasarathy S., McCullough A.J., Practice Guideline Committee of the American Association for the Study of Liver Diseases, The Practice Parameters Committee of the American College of Gastroenterology (2010). Alcoholic liver disease. Hepatology.

[5] Marroni C.A., Fleck A.M., Fernandes S.A., Galant L.H., Mucenic M., de Mattos Meine M.H., Mariante-Neto G., Brandao A.B.M. (2018). Liver transplantation and alcoholic liver disease: History, controversies, and considerations. World J. Gastroenterol..

[6] Takeuchi M., Vidigal P.T., Guerra M.T., Hundt M.A., Robert M.E., Olave-Martinez M., Aoki S., Khamphaya T., Kersten R., Kruglov E. (2021). Neutrophils interact with cholangiocytes to cause cholestatic changes in alcoholic hepatitis. Gut.

[7] Llovet J.M., Kelley R.K., Villanueva A., Singal A.G., Pikarsky E., Roayaie S., Lencioni R., Koike K., Zucman-Rossi J., Finn R.S. (2021). Hepatocellular carcinoma. Nat. Rev. Dis. Prim..

[8] Schwabe R.F., Greten T.F. (2020). Gut microbiome in HCC-Mechanisms, diagnosis and therapy. J. Hepatol..

[9] Kong L.Z., Chandimali N., Han Y.H., Lee D.H., Kim J.S., Kim S.U., Kim T.D., Jeong D.K., Sun H.N., Lee D.S. (2019). Pathogenesis, Early Diagnosis, and Therapeutic Management of Alcoholic Liver Disease. Int. J. Mol. Sci..

[10] Qu J., Ko C.W., Tso P., Bhargava A. (2019). Apolipoprotein A-IV: A Multifunctional Protein Involved in Protection against Atherosclerosis and Diabetes. Cells.

[11] Qin W., Li X., Xie L., Li S., Liu J., Jia L., Dong X., Ren X., Xiao J., Yang C. (2016). A long non-coding RNA, APOA4-AS, regulates APOA4 expression depending on HuR in mice. Nucleic Acids Res..

[12] Cheng C., Liu X.H., He J., Gao J., Zhou J.T., Fan J.N., Jin X., Zhang J., Chang L., Xiong Z. (2022). Apolipoprotein A4 Restricts Diet-Induced Hepatic Steatosis via SREBF1-Mediated Lipogenesis and Enhances IRS-PI3K-Akt Signaling. Mol. Nutr. Food Res..

[13] Peters K.E., Davis W.A., Ito J., Winfield K., Stoll T., Bringans S.D., Lipscombe R.J., Davis T.M.E. (2017). Identification of Novel Circulating Biomarkers Predicting Rapid Decline in Renal Function in Type 2 Diabetes: The Fremantle Diabetes Study Phase II. Diabetes Care.

[14] Wang P.W., Hung Y.C., Wu T.H., Chen M.H., Yeh C.T., Pan T.L. (2017). Proteome-based identification of apolipoprotein A-IV as an early diagnostic biomarker in liver fibrosis. Oncotarget.

[15] Duverger N., Tremp G., Caillaud J.M., Emmanuel F., Castro G., Fruchart J.C., Steinmetz A., Denefle P. (1996). Protection against atherogenesis in mice mediated by human apolipoprotein A-IV. Science.

[16] Recalde D., Ostos M.A., Badell E., Garcia-Otin A.L., Pidoux J., Castro G., Zakin M.M., Scott-Algara D. (2004). Human apolipoprotein A-IV reduces secretion of proinflammatory cytokines and atherosclerotic effects of a chronic infection mimicked by lipopolysaccharide. Arterioscler. Thromb. Vasc. Biol..

[17] Vowinkel T., Mori M., Krieglstein C.F., Russell J., Saijo F., Bharwani S., Turnage R.H., Davidson W.S., Tso P., Granger D.N. (2004). Apolipoprotein A-IV inhibits experimental colitis. J. Clin. Investig..

[18] Wang Y., Yang Z., Wei Y., Li X., Li S. (2021). Apolipoprotein A4 regulates the immune response in carbon tetrachloride-induced chronic liver injury in mice. Int. Immunopharmacol..

[19] Pinal-Fernandez I., Casal-Dominguez M., Derfoul A., Pak K., Miller F.W., Milisenda J.C., Grau-Junyent J.M., Selva-O’Callaghan A., Carrion-Ribas C., Paik J.J. (2020). Machine learning algorithms reveal unique gene expression profiles in muscle biopsies from patients with different types of myositis. Ann. Rheum. Dis..

[20] Gao B., Argemi J., Bataller R., Schnabl B. (2021). Serum Acylcarnitines Associated with High Short-Term Mortality in Patients with Alcoholic Hepatitis. Biomolecules.

[21] Brenner C., Galluzzi L., Kepp O., Kroemer G. (2013). Decoding cell death signals in liver inflammation. J. Hepatol..

[22] Knott C., Bell S., Britton A. (2015). Alcohol Consumption and the Risk of Type 2 Diabetes: A Systematic Review and Dose-Response Meta-analysis of More Than 1.9 Million Individuals From 38 Observational Studies. Diabetes Care.

[23] Qi W., Wang B., Yang M., Zhu L., Hu S., Sun H. (2020). The implementation of drug reposition for alcoholic hepatitis based on a sub-pathway integration strategy. Ann. Transl. Med..

[24] Im G.Y., Cameron A.M., Lucey M.R. (2019). Liver transplantation for alcoholic hepatitis. J. Hepatol..

[25] Tiberio L., Del Prete A., Schioppa T., Sozio F., Bosisio D., Sozzani S. (2018). Chemokine and chemotactic signals in dendritic cell migration. Cell. Mol. Immunol..

[26] Di Gioia M., Spreafico R., Springstead J.R., Mendelson M.M., Joehanes R., Levy D., Zanoni I. (2020). Endogenous oxidized phospholipids reprogram cellular metabolism and boost hyperinflammation. Nat. Immunol..

[27] Addanki S., Meas S., Sarli V.N., Singh B., Lucci A. (2022). Applications of Circulating Tumor Cells and Circulating Tumor DNA in Precision Oncology for Breast Cancers. Int. J. Mol. Sci..

[28] Hammoudeh S.M., Hammoudeh A.M., Bhamidimarri P.M., Al Safar H., Mahboub B., Kunstner A., Busch H., Halwani R., Hamid Q., Rahmani M. (2021). Systems Immunology Analysis Reveals the Contribution of Pulmonary and Extrapulmonary Tissues to the Immunopathogenesis of Severe COVID-19 Patients. Front. Immunol..

[29] Cheng C.W., Chang C.C., Chen H.W., Lin C.Y., Chen J.S. (2018). Serum ApoA4 levels predicted the progression of renal impairment in T2DM. Eur. J. Clin. Investig..

[30] Zhang Y., He J., Zhao J., Xu M., Lou D., Tso P., Li Z., Li X. (2017). Effect of ApoA4 on SERPINA3 mediated by nuclear receptors NR4A1 and NR1D1 in hepatocytes. Biochem. Biophys. Res. Commun..

[31] Obinata H., Kuo A., Wada Y., Swendeman S., Liu C.H., Blaho V.A., Nagumo R., Satoh K., Izumi T., Hla T. (2019). Identification of ApoA4 as a sphingosine 1-phosphate chaperone in ApoM- and albumin-deficient mice. J. Lipid Res..

[32] Soukup V., Capoun O., Pesl M., Vavrova L., Sobotka R., Levova K., Hanus T., Zima T., Kalousova M. (2019). The significance of calprotectin, CD147, APOA4 and DJ-1 in non-invasive detection of urinary bladder carcinoma. Neoplasma.

[33] Taavela J., Viiri K., Valimaki A., Sarin J., Salonoja K., Maki M., Isola J. (2021). Apolipoprotein A4 Defines the Villus-Crypt Border in Duodenal Specimens for Celiac Disease Morphometry. Front. Immunol..

[34] Ueda K., Saichi N., Takami S., Kang D., Toyama A., Daigo Y., Ishikawa N., Kohno N., Tamura K., Shuin T. (2011). A comprehensive peptidome profiling technology for the identification of early detection biomarkers for lung adenocarcinoma. PLoS ONE.

[35] Farrokhi Yekta R., Arefi Oskouie A., Rezaei Tavirani M., Mohajeri-Tehrani M.R., Soroush A.R. (2018). Decreased apolipoprotein A4 and increased complement component 3 as potential markers for papillary thyroid carcinoma: A proteomic study. Int. J. Biol. Markers.

[36] Xu M.J., Cai Y., Wang H., Altamirano J., Chang B., Bertola A., Odena G., Lu J., Tanaka N., Matsusue K. (2015). Fat-Specific Protein 27/CIDEC Promotes Development of Alcoholic Steatohepatitis in Mice and Humans. Gastroenterology.

[37] Li Y., Kang H., Chu Y., Jin Y., Zhang L., Yang R., Zhang Z., Zhao S., Zhou L. (2018). Cidec differentially regulates lipid deposition and secretion through two tissue-specific isoforms. Gene.

[38] Luo M., Lai W., He Z., Wu L. (2021). Development of an Optimized Culture System for Generating Mouse Alveolar Macrophage-like Cells. J. Immunol..

[39] Russell T., Watad A., Bridgewood C., Rowe H., Khan A., Rao A., Loughenbury P., Millner P., Dunsmuir R., Cuthbert R. (2021). IL-17A and TNF Modulate Normal Human Spinal Entheseal Bone and Soft Tissue Mesenchymal Stem Cell Osteogenesis, Adipogenesis, and Stromal Function. Cells.

[40] Kang H.S., Okamoto K., Takeda Y., Beak J.Y., Gerrish K., Bortner C.D., DeGraff L.M., Wada T., Xie W., Jetten A.M. (2011). Transcriptional profiling reveals a role for RORalpha in regulating gene expression in obesity-associated inflammation and hepatic steatosis. Physiol. Genom..

[41] Joffre J., Hellman J., Ince C., Ait-Oufella H. (2020). Endothelial Responses in Sepsis. Am. J. Respir. Crit. Care Med..

[42] Dixon L.J., Barnes M., Tang H., Pritchard M.T., Nagy L.E. (2013). Kupffer cells in the liver. Compr. Physiol..

[43] Tsutsui H., Nishiguchi S. (2014). Importance of Kupffer cells in the development of acute liver injuries in mice. Int. J. Mol. Sci..

[44] Koyama Y., Brenner D.A. (2017). Liver inflammation and fibrosis. J. Clin. Investig..

[45] Yang W., Tao Y., Wu Y., Zhao X., Ye W., Zhao D., Fu L., Tian C., Yang J., He F. (2019). Neutrophils promote the development of reparative macrophages mediated by ROS to orchestrate liver repair. Nat. Commun..

[46] Calvente C.J., Tameda M., Johnson C.D., Del Pilar H., Lin Y.C., Adronikou N., De Mollerat Du Jeu X., Llorente C., Boyer J., Feldstein A.E. (2019). Neutrophils contribute to spontaneous resolution of liver inflammation and fibrosis via microRNA-223. J. Clin. Investig..

[47] Shen M., Shen Y., Fan X., Men R., Ye T., Yang L. (2020). Roles of Macrophages and Exosomes in Liver Diseases. Front. Med..

[48] Schuppan D. (2015). Liver fibrosis: Common mechanisms and antifibrotic therapies. Clin. Res. Hepatol. Gastroenterol..

[49] Newman A.M., Liu C.L., Green M.R., Gentles A.J., Feng W., Xu Y., Hoang C.D., Diehn M., Alizadeh A.A. (2015). Robust enumeration of cell subsets from tissue expression profiles. Nat. Methods.

