# Supplementary material for: Apolipoprotein A-IV Has Bi-Functional Actions in Alcoholic Hepatitis by Regulating Hepatocyte Injury and Immune Cell Infiltration
Source: Int J Mol Sci. 2022 Dec 30;24(1):670. doi: 10.3390/ijms24010670 (PMC9820766; doi:10.3390/ijms24010670)
Supplement: Supplementary file 1 [file ijms-24-00670-s001.zip › ijms-1980349-supplementary.pdf]

Supplement Figures

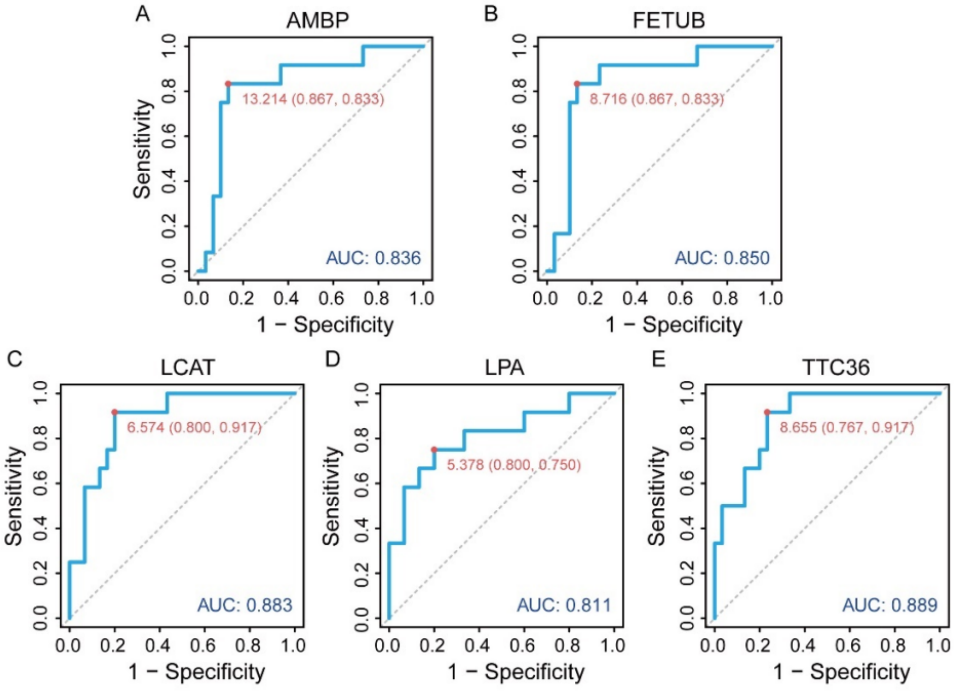

Supplementary Figure S1. The ROC curves of five hub genes.

(A) *AMBP*, (B) *FETUB*, (C) *LCAT*, (D) *LPA*, and (E) *TTC36* in the ArrayExpress cohort.

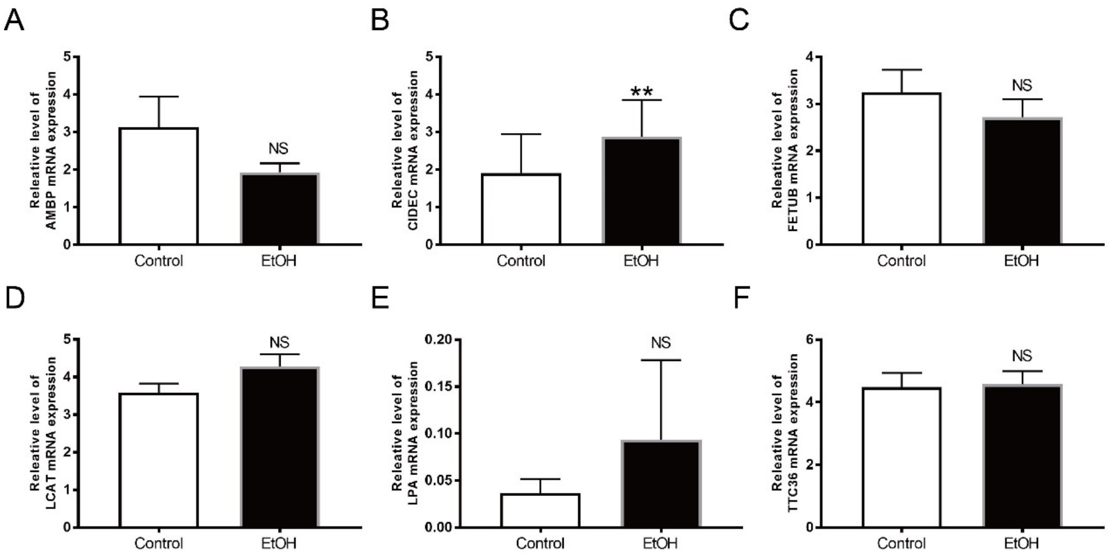

Supplementary Figure S2. Expression of AMBP, CIDEC, FETUB, LCAT, LPA, and TTC36 in AH and normal liver tissues in mice.

(A) AMBP, (B) CIDEC, (C) FETUB, (D) LCAT, (E)LPA, and (F) TTC36. ***P* < 0.01 vs. the control group

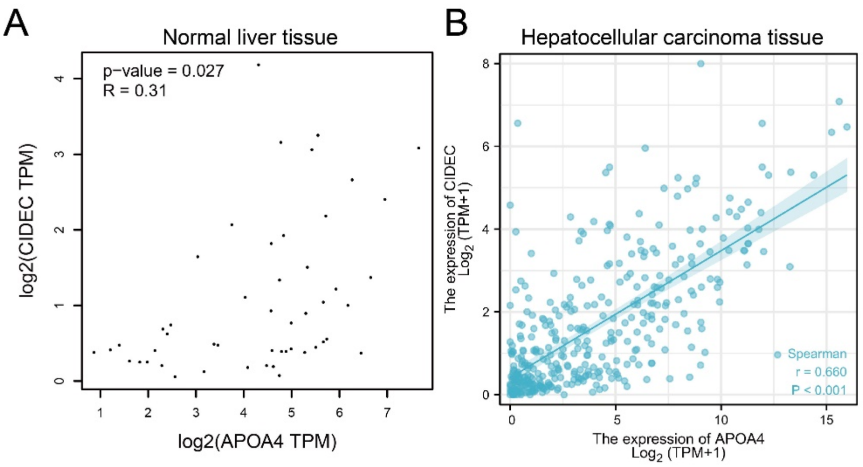

Supplementary Figure S3. Correlation between *APOA4* and *CIDEC* in normal liver tissue and hepatocellular carcinoma.

(A) Correlation between APOA4 and *CIDEC* in normal liver tissue, based on GEPIA datasets, (B) Correlation between APOA4 and *CIDEC* in hepatocellular carcinoma tissue, based on TCGA datasets.

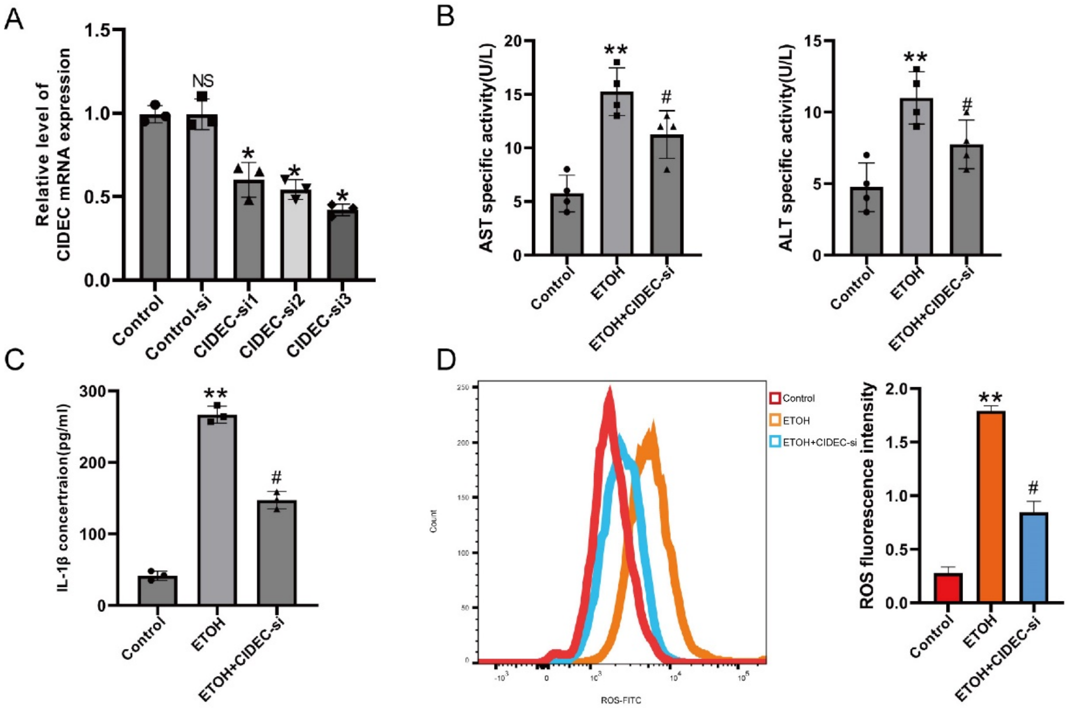

Supplementary Figure S4. CIDEC knockdown inhibited cell injury in ETOH-treated AML-12 cells.

(A) AML-12 cells were transfected with CIDEC siRNA or control siRNA for 48 h. The expression of CIDEC was determined with qRT-PCR (n = 3). (B) The AST and ALT activity in AML-12 cells after treatment with CIDEC-si stimulated by with/without ETOH. (C) The activity of IL-1β detected by ELISA kit. (D) ROS fluorescence intensity was detected by flow cytometry. (n = 3) *$P < 0.05$, **$P < 0.01$ *vs* the control group; $^{\#}P < 0.05$ *vs* the ETOH group or $^{NS}P > 0.05$ *vs* the Control group.

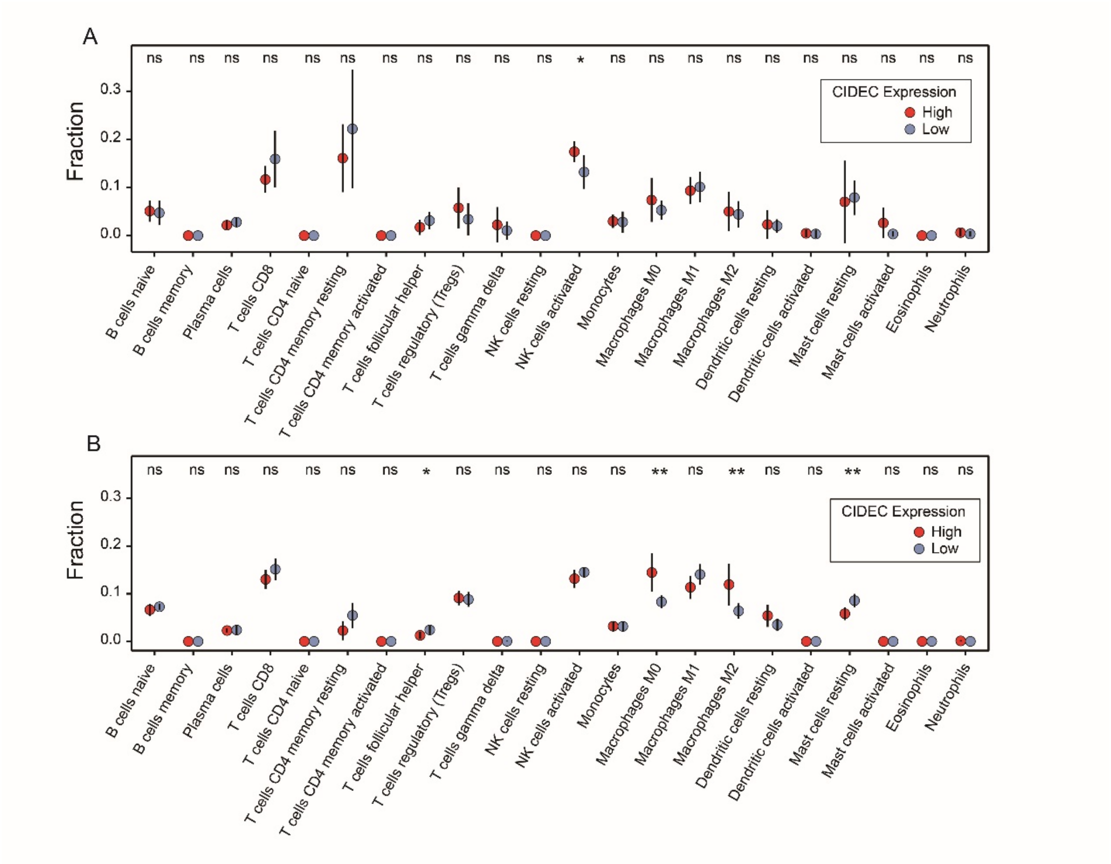

Supplementary Figure S5. The proportion of 22 subpopulations of immune infiltrating cells in normal liver tissues and AH tissues was connected with *CIDEC*.

(A) Correlation of *CIDEC* and immune infiltrating cells in normal liver tissue because of Cibersort. (B) *CIDEC* and immune infiltrating cells in AH tissue correlate, based on Cibersort. $^{*}P < 0.05$, $^{**}P < 0.01$ *vs* the high group.
